# A Silicon Resonant Pressure Microsensor Based on Frequency-Ratio Measurement for High-Temperature Applications

**DOI:** 10.3390/mi17030293

**Published:** 2026-02-27

**Authors:** Zhaoyuan Tan, Pengxiang Ye, Xiaohan Liu, Bo Xie, Yulan Lu, Deyong Chen, Junbo Wang

**Affiliations:** 1 State Key Laboratory of Transducer Technology, Aerospace Information Research Institute, Chinese Academy of Sciences, Beijing 100190, China; tanzhaoyuan23@mails.ucas.ac.cn (Z.T.); yepengxiang22@mails.ucas.ac.cn (P.Y.); liuxiaohan23@mails.ucas.ac.cn (X.L.); luyl@aircas.ac.cn (Y.L.); dychen@mail.ie.ac.cn (D.C.); 2 School of Electronic, Electrical and Communication Engineering, University of Chinese Academy of Sciences, Beijing 100049, China

**Keywords:** high-temperature and high-pressure, microelectromechanical system, resonant pressure sensor, frequency measurement

## Abstract

This paper presents a high-temperature silicon resonant pressure microsensor capable of stable operation up to 175 °C and 175 MPa, addressing the critical need for reliable pressure monitoring in deep well drilling and petroleum exploration. To overcome the inherent trade-off between pressure range and sensitivity in diaphragm-based sensors, the sensor incorporates V-shaped micro-beam supports that convert radial compressive stress into supplementary axial tensile stress on the resonant beams. This innovative force-transmission structure enhances both pressure resistance and positive stress sensitivity, enabling range extension while maintaining adequate sensitivity. A key feature of this work is the implementation of a frequency-ratio measurement scheme utilizing a dedicated pressure-insensitive reference resonator. This approach effectively eliminates the dependence on the stability of the external crystal oscillator frequency, a significant source of error in high-temperature environments where stable clock sources are costly or unavailable. Experimental results demonstrate that the fabricated sensor achieves a pressure sensitivity of 723.56 ppm/MPa for Resonator I and −436.60 ppm/MPa for Resonator II. The frequency-ratio output scheme maintains a measurement accuracy better than 0.02% FS (within the 0–36 MPa verification range) even when using a low-stability oscillator at 125 °C, significantly outperforming conventional direct-frequency measurement methods. The sensor’s combination of an extended pressure range, high-temperature capability, and robust frequency-ratio output offers a promising solution for high-precision pressure sensing in extreme downhole conditions.

## 1. Introduction

In petroleum exploration and development, real-time bottom-hole pressure measurements are essential for dynamically adjusting drilling fluid density to maintain pressure within the safe window between formation pore pressure and fracture pressure, thereby preventing kicks and blowouts caused by underbalanced pressure as well as lost circulation and formation fracturing caused by overpressure in deep and ultra-deep drilling operations [1,2,3]. With the continuous growth of global energy demand, oil and gas exploration is advancing into deep and ultra-deep formations, imposing stricter requirements on the operating environment and pressure range of pressure sensors [4,5,6]. Meanwhile, the reduction in drill bit diameter in deep drilling and the challenge of narrow density windows also poses demands for the miniaturization and high precision of sensors. Currently, the burial depth of some typical oil and gas fields in China reaches 8000 m, and the downhole environment is harsh with high temperature (>150 °C) and high pressure (>175 MPa). This imposes requirements on the pressure resistance of sensors and the high-temperature stability of measurement circuits.

For piezoresistive sensors, some studies have adopted the volume compression principle and miniaturized cavity structures to achieve stress concentration, thereby resolving the conflict between range extension and sensitivity [7,8,9]. However, due to temperature drift and other interference factors, such sensors still exhibit limited full-range accuracy. Quartz resonant pressure sensors are widely used in petroleum exploration due to their high accuracy [10,11,12]. However, their large size, complex fabrication, and high cost hinder the miniaturization and large-scale deployment of measurement equipment.

In contrast, silicon-based pressure sensors possess the advantages of small size and low cost, making them more conducive to the development of miniaturization for measurement-while-drilling (MWD) sensors. However, silicon resonant sensors exhibit issues such as significant temperature drift, a trade-off between device pressure resistance and sensitivity, and difficulty in high-precision frequency acquisition when operating in downhole high-temperature and high-pressure environments.

To address the resonant frequency drift of the sensor caused by changes in the Young’s modulus and the thermal expansion coefficient of silicon under high temperatures, the adoption of a dual-beam differential scheme enables temperature self-compensation and reduces frequency drift. Range of a resonant pressure sensor is often constrained by the diaphragm-based sensing principle. Qian et al. proposed a silicon resonant high-pressure sensor based on a composite pressure-sensitive mechanism that integrates diaphragm bending and volume compression [13]. By reducing the diaphragm area and using the differential dual resonator, this approach effectively balances pressure resistance and sensitivity, which achieves high accuracy and stability up to 100 MPa. However, due to the constraint of the diaphragm area, further range expansion remains challenging.

In high-temperature measurement, existing resonant pressure sensors characterize pressure via output frequency. In practical applications, they are required to achieve digital output of either frequency or calculated pressure; thus, the accuracy of digital frequency measurement directly affects the precision of pressure measurement. Currently, common frequency measurement schemes largely rely on the stability of reference crystal oscillators or clock frequencies [14,15,16]. However, most temperature-compensated crystal oscillators (TCXOs) with high stability (less than 1 ppm) have an operating temperature range of less than 85 °C [17], failing to meet the requirements of downhole high-temperature environments (above 125 °C). Although high-temperature crystal oscillators capable of withstanding high-temperature environments have a wider operating temperature range, they suffer from poor stability (≥40 ppm, up to 250 ppm) [18,19] and high cost—posing significant challenges to the sensor’s measurement precision and the cost of control circuits. EerNisse et al. proposed a three-crystal sensor structure, using a reference crystal made of SC-cut quartz, combined with a quartz pressure sensor and a quartz temperature sensor [10,20]. By mixing the reference crystal output signal with pressure and temperature signals, frequency computation is achieved, reducing counting requirements. However, this scheme requires three quartz crystals with different tangential cuts and shapes, resulting in high manufacturing difficulty and large space occupation. Additionally, under sudden temperature changes, temperature gradients may exist between the crystals, affecting measurement precision.

To address these challenges, this study proposes a volume compression-based high-temperature and high-pressure sensitive chip integrated with V-shaped positive–negative stress resonators and a reference resonator, along with a corresponding testing method, to tackle the three issues of pressure resistance, temperature compensation, and frequency measurement.

## 2. Sensor Structure and Working Principle

**Figure 1:**
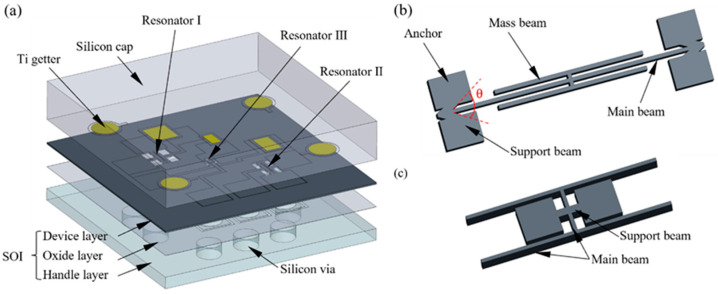
Schematic diagram of sensor structure model (**a**) overall sensor architecture. (**b**) Pressure-sensitive resonators I and II with V-shaped supports. (**c**) Reference resonator III with axial isolation.

The sensor structure is illustrated in Figure 1a. A silicon wafer serves as the cap layer, while a silicon-on-insulator (SOI) wafer is used to fabricate the device and handle layers. The resonant beams are located in the device layer of the SOI wafer and form suspended structures after releasing the buried oxide layer. To meet high-temperature measurement requirements, electrostatic excitation and capacitive detection are adopted, with corresponding electrodes distributed within the device layer. Getter materials are placed in the resonator and getter chambers on the silicon cap to achieve high-vacuum packaging. The sensor incorporates three resonant beams, each positioned on diaphragms of different sizes. The entire handle layer acts as the primary diaphragm, while the diaphragm structures are formed by combining the device layer with the resonator regions of the cap layer. Resonators I and II are pressure-sensitive resonator beams (Figure 1b) with positive and negative pressure sensitivities, respectively, used for differential pressure detection. Resonator III serves as a reference resonator beam (Figure 1c) that is nearly pressure-insensitive, and its resonant frequency is used for high-temperature frequency compensation.

For the pressure-sensitive beams (I and II), the axial stress can be written as

(1) $$\sigma =\sigma_{comp}+\sigma_{tens}$$

where $\sigma_{comp}$ denotes the compressive component induced by volumetric compression under high pressure; this value is only related to the ambient pressure and is not affected by the diaphragm structure, and $\sigma_{tens}$ is the tensile component arising from diaphragm bending, whose magnitude can be adjusted by modifying the dimensions of the diaphragm structure. Thus, by tailoring the diaphragm geometry, the relative magnitudes of these components at a given pressure can be tuned to realize matched positive/negative stresses for the differential pair.

To enhance overpressure tolerance, the diaphragm area must be reduced; however, this also shortens the available resonator length and reduces bending-induced tensile stress. To supplement axial pressure, V-shaped micro-beam supports are introduced at both ends of the resonant beam to convert tangential compressive stress into axial tensile stress; the axial supplementary stress at this point is:

(2) $$\sigma_{ts}=\frac{1}{2}\sigma_{tan}\sin\theta$$

where $\sigma_{tan}$ is the tangential compressive stress on the resonator and θ is the included angle between the pair of V-supports (Figure 1b). The total axial stress becomes

(3) $$\sigma =\sigma_{comp}+\sigma_{tens}+\sigma_{ts}$$

When a resonant beam is subjected to axial stress, its resonant frequency is given by

(4) $$f_{0}\left(\sigma \right)=f_{0}\sqrt{1+\frac{\sigma }{\sigma_{E}}}$$

where $f_{0}$ is the stress-free natural frequency and $\sigma_{E}=\frac{7E}{2}{\left(\frac{b}{l}\right)}^{2}$ is the Euler-type critical stress of the beam (with Young’s modulus E, beam width b, and length l). For the two pressure-sensitive resonant beams, by adjusting the diaphragm dimension parameters, the axial stresses of the two beams are made to exhibit opposite polarities (one positive and one negative) under the same pressure.

To mitigate the increase in base frequency caused by the reduction in resonator length, which subsequently affects the adaptation of the closed-loop control system, a mass block of appropriate weight is introduced at the center of the resonant beam. This mass block counterbalances the frequency shift resulting from the shortening of the resonator. Considering the subsequent resonator release process and driving control requirements, an “H”-shaped mass beam is positioned at the center of the resonator. The width of the mass beam is designed to be similar to that of the resonant beam, and its length is aligned with the driving electrode, facilitating subsequent release and drive operations. For simplified calculation, the “H”-shaped mass beam is approximated as a lumped mass at the center of the resonant beam. The first-order resonant frequency of the resonant beam is thus expressed as:

(5) $$f_{0}=\frac{1}{2\pi }\sqrt{\frac{\frac{256}{15}Ehb^{3}}{\left(M+\frac{128}{315}m_{b}\right)l^{3}}}$$

where E is Young’s modulus, h is the height of the device layer, b is the width of the main beam, M is the total weight of the mass beam, $m_{b}$ is the weight of the main beam, and l is the length of the resonant beam.

Resonator III is designed to be nearly pressure insensitive. External stress is routed from the anchors into a transverse support beam and distributed along its axis; a longitudinal isolation beam decouples this path from the resonant beam, rendering the resonance largely insensitive to pressure. Mechanical coupling between paired beams through the longitudinal member improves the quality factor and stabilizes the frequency.

## 3. Sensor Simulation and Design

**Figure 2:**
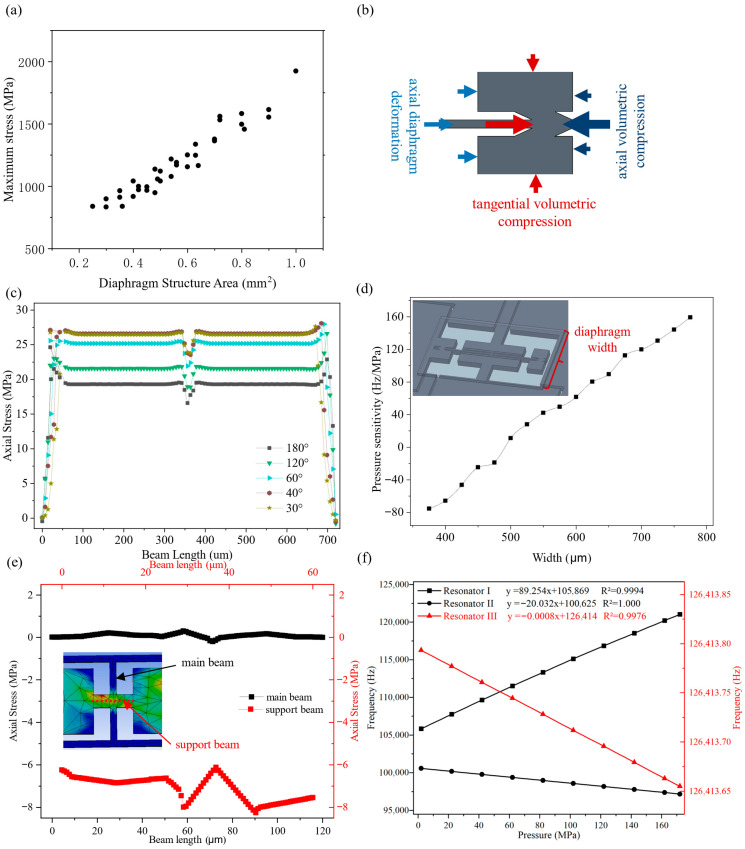
(**a**) Maximum stress under different diaphragm structure areas. (**b**) V-shaped support beams anchor points stress distribution schematic diagram. (**c**) Axial stress versus V-support angle for a pressure-sensitive resonator. (**d**) Pressure sensitivity under different diaphragm widths. (**e**) Axial-stress distribution along the support beams and main beam of the reference resonator. (**f**) Simulated frequency–pressure characteristics (three resonators).

According to the finite element analysis (FEA) simulation results shown in Figure 2a, an excessively large diaphragm area will cause the device stress to exceed the allowable stress threshold, significantly increasing the risk of structural failures such as diaphragm rupture and resonant beam deformation. After multiple rounds of iterative simulations and parameter adjustments, the basic geometric parameters of the diaphragm structure in the sensor core were finally determined. For the two pressure-sensitive resonant beams (Resonator I and Resonator II), a uniform resonant beam length (720 µm) was selected, matched with a resonant cavity length of 770 µm; meanwhile, to ensure structural stability under pressure, the resonant cavity width was strictly limited to less than 700 µm.

To optimize the stress conversion efficiency of the V-shaped support beams as illustrated in Figure 2b, with the diaphragm width and anchor position fixed as control variables, the variation law of the axial tensile stress of the resonant beams with the angle of the V-shaped support beams was systematically investigated through FEA. As shown in Figure 2c, within the simulated angle range, the axial tensile stress of the resonant beams exhibits a positive correlation with the decrease in the angle of the V-shaped support beams. The reason for this is that a smaller support beam angle can more effectively redistribute the stress generated by diaphragm deformation, concentrating the stress on the resonant beams rather than dissipating it in the support structure. After comprehensively considering the target stress magnitude and subsequent process constraints, the width and angle of the V-shaped support beams were finally determined to be 20 µm and 56°.

On the basis of fixing the geometric dimensions of the resonant beams, and in accordance with the theory presented earlier, the regulation of the pressure sensitivity of the pressure-sensitive resonant beams can be achieved by adjusting the diaphragm structure width. Therefore, the relationship between the pressure sensitivity of the resonant beams under different diaphragm structure widths was obtained through finite element analysis (FEA), and the results are shown in Figure 2d. It can be observed that within the tested width range, the pressure sensitivity of the resonant beams exhibits a linear increasing trend from negative to positive as the diaphragm width increases, which can meet the requirement of differential positive and negative pressure sensitivities for the dual beams in the design. With reference to the pressure resistance performance of the device, two optimal diaphragm width values were finally determined: 685 µm and 484 µm. These two widths not only provide sufficient axial stress to ensure sensitivity but also maintain structural integrity under high pressure.

For Resonator III, which serves as the reference for output frequency, the design requires it to be stress insensitive. To this end, a support beam structure perpendicular to the axial direction of the resonant beam was adopted, ensuring that the stress generated by anchor deformation is mainly distributed on the support beams. This stress is orthogonal to the axial direction of the resonant beam, making the axial stress of the resonant beam close to zero. Meanwhile, a dual-beam coupling structure was introduced to improve the stability and anti-interference capability of this resonant beam. From the simulation results illustrated in Figure 2e, this structure achieves effective isolation between the resonant beam and external interference stress, ensuring the stability of the resonant frequency.

Based on the above simulation results, the key dimensional parameters of the sensor were finally determined as shown in Table 1. To avoid mutual frequency interference between the two resonators during operation, differentiated adjustments were made to the mass beam widths of Resonator I and Resonator II.

Figure 2f presents the simulated frequency–pressure characteristics of the three resonators (Resonator I, II, and III) under the determined dimensions, which intuitively reflects the sensitivity performance of the sensor. At these operating conditions, the maximum simulated stress is 1321 MPa at the center of the diaphragm’s long edge, below yield strength of silicon (7 GPa), and subsequent high-pressure testing revealed no structural failures, validating the reliability of the sensor core.

## 4. Microfabrication Process and Packaging

**Figure 3:**
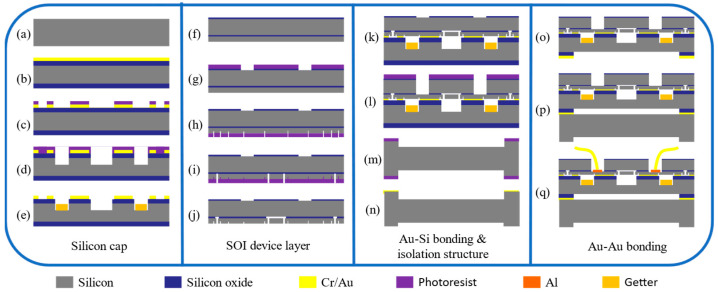
Process flow of the sensor fabrication. (**a**–**c**) Patterning of gold layer on bonding surface. (**d**) Patterned etching of resonant cavity and getter cavity. (**e**) Getter evaporation. (**f**,**g**) Patterning of handle layer. (**h**,**i**) Structural patterning of device layer. (**j**) Resonant beam release. (**k**,**l**) Au–Si bonding and electrode hole etching. (**m**–**o**) Patterning of isolation structure and gold layer evaporation. (**p**,**q**) Au–Au thermocompression bonding and wire bonding.

The fabrication of the sensor chip involves three wafers in total, and the fabrication and packaging of the sensitive structure are achieved through two bonding steps. The schematic diagram of the specific fabrication process is shown in Figure 3.

A 1000 µm-thick silicon wafer is selected and thermally oxidized to form an insulating oxide layer (Figure 3a). As shown in Figure 3a,b, a Cr/Au (500/5000 Å) bonding layer was deposited on the wafer surface by physical vapor deposition (PVD) using an electron-beam evaporator (BJD-2000, Ferrotec, Nihonbashi, Japan). As shown in Figure 3c–e, the metal layer was patterned by wet etching (a mixed solution of I_2_ and KI for the Au layer, a mixed solution of KMnO_4_ and NaOH for the Cr layer), followed by sequential dry etching of the oxide and silicon using a dielectric film etcher (GSE C200, NAURA, Beijing, China) and a deep-silicon etcher (HSE 200, NAURA, China) to form 20 µm-deep resonator and getter cavities. Ti/Au getter material (1 μm/300 Å) was then deposited into these cavities by PVD through a laser-cut glass shadow mask, and the getter was activated at 450 °C during the subsequent Au–Si eutectic bonding step as diffusion of the Au capping layer exposed Ti and restored the sorption capability.

For the SOI wafer (40 µm device layer/2 µm oxide layer/300 µm handle layer), backside alignment marks were defined by dry etching (Figure 3f,g), and frontside etching formed the device-layer structures together with trenches (5 µm deep) to prevent Au overflow (Figure 3h,i). HF vapor was used to remove part of the buried oxide layer, releasing the resonant beams (Figure 3j). The SOI device layer was bonded to the silicon cap by Au–Si eutectic bonding using a wafer bonder (SB6e, SUSS, Garching, Germany) at 5000 mbar and 390 °C (Figure 3k). And silicon via structures were subsequently etched to provide electrical feedthroughs (Figure 3l).

A 2 mm-thick silicon wafer was dry etched to form a stress-isolation structure with etch depths of 200 µm at the bonding surface and 300 µm at the bottom (Figure 3m), which prevents direct contact between the sensitive die and the metal housing and buffers assembly induced thermal stress arising from the mismatch in coefficients of thermal expansion. After depositing Cr/Au (500/5000 Å) bonding layers on both wafers by PVD, Au–Au thermocompression bonding (5000 mbar, 350 °C) was performed to bond the sensor core to the isolation layer (Figure 3n–p).

**Figure 4:**
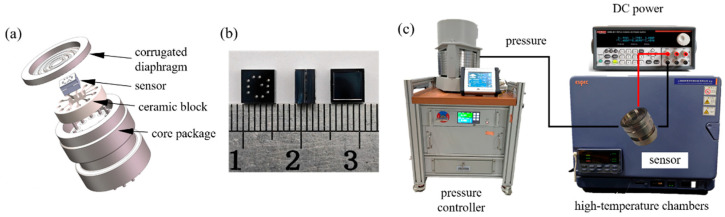
(**a**) Pressure-tight package schematic. (**b**) Sensor core. (**c**) High-temperature/high-pressure test setup.

After the wafer is fabricated, it is diced into individual chips and packaged. Figure 4b shows the completed sensor chip, which is electrically connected to the packaging pins using gold wire bonding. For high-temperature and high-pressure testing, the sensor chip is packaged in a specialized high-pressure measurement fixture (Figure 4a). This fixture includes a can-shell base, ceramic block, and corrugated diaphragm that transmits external pressure to the sensor core. Metal pins are used for signal extraction, and the ceramic block enhances pressure resistance by reducing diaphragm deformation.

## 5. Sensor Testing

The test system is shown in Figure 4c. A high-pressure hydraulic piston gauge (PG7202, Fluke, Everett, WA, USA) supplies pressure up to 175 MPa with 0.005% full scale accuracy. A small-scale high-temperature chamber (STH-120, ESPEC, Osaka, Japan) maintains temperatures from 50 °C to 175 °C with ±0.5 °C stability. A DC power supply powers the sensor, and a dedicated high-temperature control circuit conditions the resonator signals.

**Figure 5:**
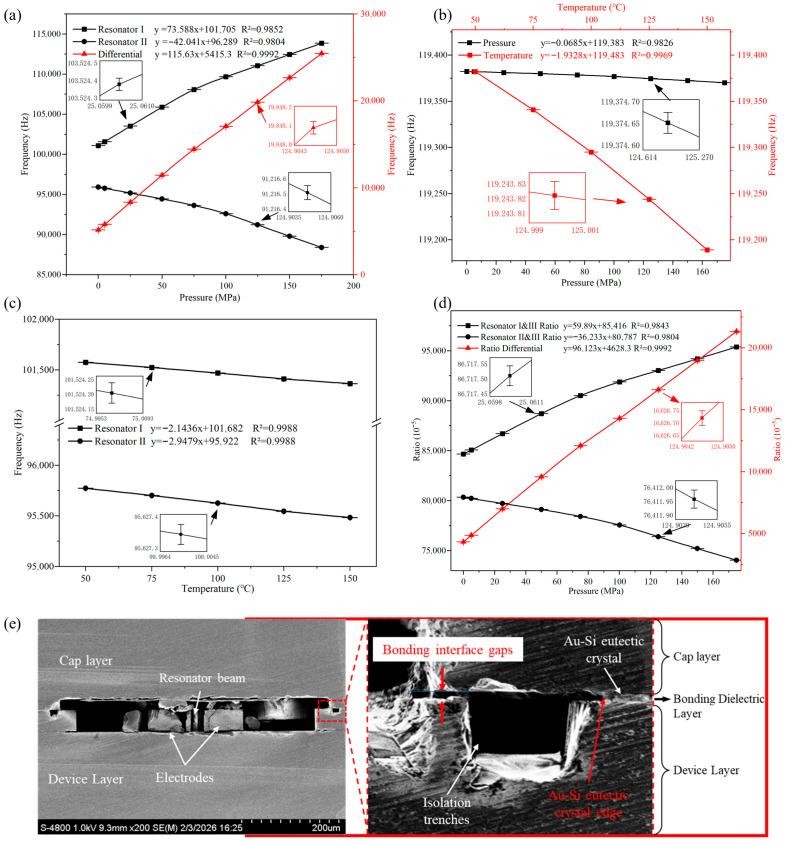
(**a**) Pressure characteristics of pressure-sensitive resonators (resonators I, II). (**b**) Temperature/pressure characteristics of the reference resonator (resonator III). (**c**) Temperature characteristics of pressure-sensitive resonators (resonators I, II). (**d**) Pressure characteristics using the frequency-ratio output. (**e**) SEM micrograph of the resonant cavity cross-section.

Figure 5a–c illustrates the frequency characteristic curves of the sensor’s Resonators I, II, and III under varying temperature and pressure conditions, with each data point obtained from five repeated tests and error bars representing the standard deviation. Test data show that Resonator I has a pressure sensitivity of approximately 73.588 Hz/MPa (723.56 ppm/MPa) and a temperature sensitivity of roughly −2.14 Hz/°C (−21.05 ppm/°C); Resonator II exhibits a pressure sensitivity of about −42.041 Hz/MPa (−436.60 ppm/MPa) and a temperature sensitivity of around −2.95 Hz/°C (−30.75 ppm/°C); in contrast, Resonator III features a pressure sensitivity of approximately 0.0685 Hz/MPa (0.57 ppm/MPa) and a temperature sensitivity of roughly −1.93 Hz/°C (−16.15 ppm/°C).

Tests found that the pressure-sensitive beams had poor linearity in the 75–100 MPa pressure range, with a noticeable change in pressure sensitivity. Nevertheless, good linearity can be obtained through differential post-processing of the two beams. This behavior is attributed to bonding interface gaps that amplify the diaphragm effect. As can be seen from Figure 5e, the Au–Si eutectic crystal edge is at a certain distance from the resonant cavity edge, which results in the presence of gaps between the cap layer and the device layer. When the two surfaces contact each other under high pressure, the dimensions of the membrane structure gradually return to the designed state, and the pressure sensitivities of the resonant beams recover to a matched condition.

Compared with the simulated results in Figure 2f, the resonant frequencies of all three resonators exhibit deviations of approximately 4–6 kHz, primarily attributed to differences between the simulated and actual material parameters. However, these deviations do not affect sensor performance. The pressure sensitivities of the two pressure-sensitive resonators (Resonators I and II) differ significantly from the simulated values, mainly because the simulation did not account for the bonding interface gap effects described earlier. Nevertheless, within the 0–75 MPa range (where the bonding interface gaps still exist, consistent with the simulation conditions), the measured pressure sensitivities are 93.67 Hz/MPa and −30.44 Hz/MPa, respectively, which are reasonably close to the simulated values (89.25 Hz/MPa and −20.03 Hz/MPa). Although the individual sensitivities deviate from the simulated values, the measured differential sensitivity of the two resonators (124.11 Hz/MPa) remains close to the simulated value (109.29 Hz/MPa) across the full pressure range and is sufficiently large for effective differential measurement, with both resonators maintaining monotonic pressure responses that satisfy the design requirements. The remaining deviations in this range are primarily attributed to fabrication tolerances (diaphragm width, Au–Si eutectic crystal width around the resonant cavity, bonding alignment deviations, etc.). For the reference resonator (Resonator III), the pressure sensitivity is approximately 0.068 Hz/MPa higher than the simulated value, also due to fabrication tolerances, yet this still satisfies the requirements for frequency-ratio measurement.

For the reference resonant beam, it exhibits extremely low pressure sensitivity and thus can be considered insensitive to pressure variations—consistent with the requirements of subsequent tests. When the sensor adopts the frequency-ratio output scheme, its ratio characteristic curve under different pressures is illustrated in Figure 5d. As shown in the figure, the pressure sensitivities of Resonators I and II are 701.16 ppm/MPa and −448.50 ppm/MPa, respectively, which are highly consistent with the test results of frequency–pressure sensitivity. This validates the stability and accuracy of the sensor under both the differential measurement and frequency-ratio output schemes.

## 6. Pressure Computation Scheme

In terms of frequency measurement and calculation, the measurement accuracy of resonant pressure sensors depends on the high-precision acquisition of resonant frequencies. For example, the frequency measurement error of the commonly used equal-precision frequency measurement method is affected by the stability of the clock signal. Traditional schemes usually use high-precision temperature-compensated crystal oscillators (TCXOs) as clock signals; however, only low-stability crystal oscillators can be used under high temperatures, and their frequency drift will significantly increase the calculation error. To address this issue, a stress-insensitive reference beam III is integrated into the sensor core, and a frequency-ratio calculation scheme is proposed. This scheme takes the ratios of the frequencies of the sensitive beams to the frequency of the reference beam as variables for pressure calculation, which theoretically offsets crystal oscillator drift and reduces the requirement for crystal oscillator stability. The specific principal analysis is as follows:

For the three resonators (I–III), the pressure–temperature mapping can be expressed as

(6) $$\left\{\begin{array}{c} f_{1}=F_{1}(P,T)\\ f_{2}=F_{2}(P,T)\\ f_{3}=F_{3}(T) \end{array}\right.$$

where P represents the pressure measured by the sensor, T is the ambient temperature of the sensor, $f_{1}$, $f_{2}$, and $f_{3}$ are the resonant frequencies of Resonators I, II, and III, respectively, and $F_{1}$, $F_{2}$, and $F_{3}$ are the corresponding functions that relate the resonant frequencies to pressure and temperature.

**Figure 6:**
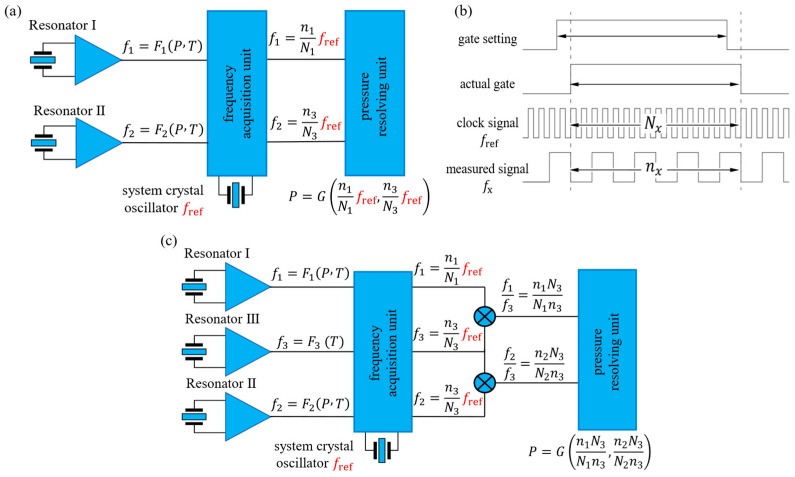
(**a**) Schematic diagram of direct pressure computation scheme using dual-beam frequencies. (**b**) Schematic diagram of multi-period counting method. (**c**) Schematic diagram of pressure computation scheme using tri-beam frequency ratios.

As shown in Figure 6a, the commonly used multi-period counting method is employed to measure the frequencies of the resonant beams [21]. At this point, the frequency acquisition module counts the cycles of the square-wave signals from the three resonators and the reference clock within the same measurement window:

(7) $$f_{x}=\frac{n_{x}}{N_{x}}f_{ref}\;x\in \{1,2,3\}$$

where $n_{x}$ is the cycle count of the resonant beam, $N_{x}$ is the cycle count of the crystal oscillator, and $f_{ref}$ is the nominal frequency of the crystal oscillator.

The frequencies of the three resonant beams acquired by the frequency acquisition module can be expressed as

(8) $$\left\{\begin{array}{c} f_{1}=\frac{n_{1}}{N_{1}}f_{ref}=F_{1}\left(P,T\right)\\ f_{2}=\frac{n_{2}}{N_{2}}f_{ref}=F_{2}\left(P,T\right)\\ f_{3}=\frac{n_{3}}{N_{3}}f_{ref}=f_{3}=F_{3}\left(T\right) \end{array}\right.$$

As shown in Equation (8) and Figure 6b, if $f_{1}$ and $f_{2}$ are used directly for pressure calculation, fluctuations in $f_{ref}$ will introduce measurement errors. To eliminate this influence, frequency ratios are used instead:

(9) $$\left\{\begin{array}{c} \frac{f_{1}}{f_{3}}=\frac{n_{1}N_{3}}{N_{1}n_{3}}=\frac{F_{1}\left(P,T\right)}{F_{3}\left(T\right)}\\ \frac{f_{2}}{f_{3}}=\frac{n_{2}N_{3}}{N_{2}n_{3}}=\frac{F_{2}\left(P,T\right)}{F_{3}\left(T\right)} \end{array}\right.$$

Pressure and temperature can be solved as follows:

(10) $$\left\{\begin{array}{c} P=G\left(\frac{n_{1}N_{3}}{N_{1}n_{3}},\frac{n_{2}N_{3}}{N_{2}n_{3}}\right)\\ T=H\left(\frac{n_{1}N_{3}}{N_{1}n_{3}},\frac{n_{2}N_{3}}{N_{2}n_{3}}\right) \end{array}\right.$$

As shown in Equation (10) and Figure 6c, $f_{ref}$ is entirely eliminated. The calculation results depend solely on the frequency counting results, such that the calculation results are no longer affected by fluctuations in the crystal oscillator frequency.

**Figure 7:**
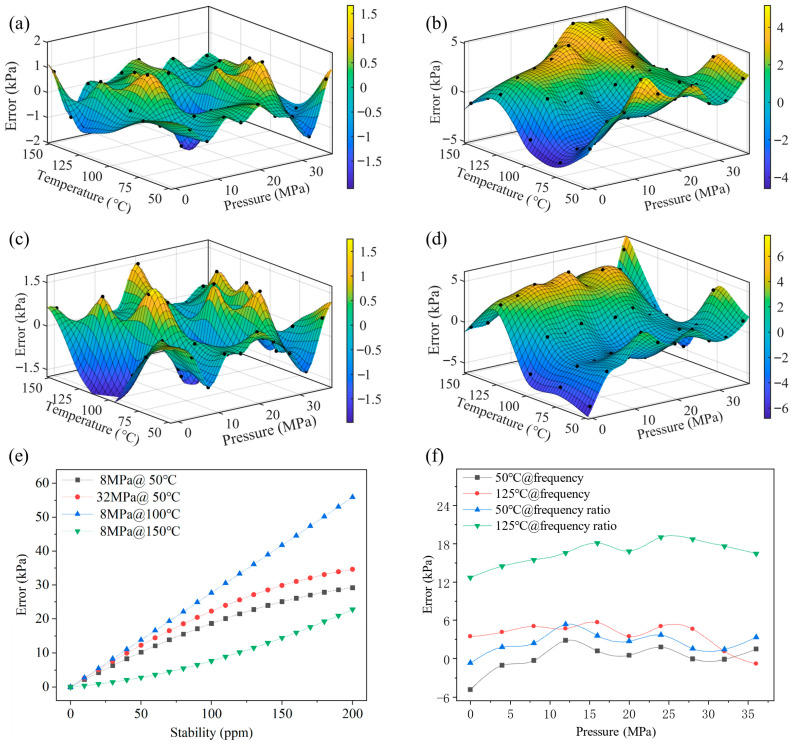
(**a**) Fit error using direct-frequency regressors. (**b**) Validation error using direct-frequency regressors. (**c**) Fit error using ratio-metric regressors. (**d**) Validation error using ratio-metric regressors. (**e**) Theoretical pressure-error versus oscillator-stability curves at selected pressures. (**f**) Comparison of the two computation schemes with high-temperature crystal oscillators.

In high-temperature environments (>150 °C), crystal oscillators typically exhibit poor stability (≥40 ppm, up to 250 ppm). According to Equation (8), oscillator drift introduces frequency acquisition errors, affecting pressure calculation. Figure 7e shows the theoretical pressure error vs. oscillator stability. When oscillator error reaches 30 ppm, the pressure error exceeds 0.02% FS, significantly impacting accuracy.

To assess the performance of two pressure fitting methods—fitting using single-frequency data versus fitting using the frequency-ratio of the reference resonant beam—a frequency acquisition circuit integrated with a high-precision temperature-compensated crystal oscillator (BT0507BH3I287DN12.8, XTALTQ, Chengdu, China, stability of 0.5 ppm) was employed for frequency acquisition. The closed-loop output frequencies of the sensor’s three resonant beams were measured and recorded outside the temperature chamber. The pressure range was set to 0–36 MPa with a step size of approximately 4 MPa, while the temperature range spanned 50–150 °C with a step size of 25 °C.

Using only the frequency data of Resonator I and Resonator II, a binary fifth-order polynomial fitting scheme was employed for fitting and verification of the sensor [22], as shown in Figure 7a, the sensor achieves a fitting accuracy of 0.01% FS (within the 0–36 MPa verification range). Subsequent verification results are presented in Figure 7b, the sensor’s measurement accuracy is better than 0.02% FS. When frequency-ratios were used for fitting, the sensor also achieved 0.01% FS fitting accuracy (Figure 7c) and better than 0.02% FS measurement accuracy (Figure 7d). Figure 7e shows the theoretical pressure error versus oscillator stability, simulated using the dual-frequency fitting coefficients. When oscillator drift reaches 30 ppm, the pressure error exceeds 0.02% FS, indicating that direct-frequency measurement will fail under high-temperature conditions where crystal oscillators typically exhibit poor stability. These results demonstrate that when the crystal oscillator exhibits sufficiently high frequency stability, using frequency-ratio data for calculation and fitting achieves the same level of accuracy as using single-frequency data, demonstrating that both schemes can achieve high-precision pressure calculation under normal temperature conditions.

To compare the two schemes under poor oscillator stability, the crystal oscillator was replaced with a conventional high-temperature crystal oscillator (CX3225SA, KYOCERA, Kyoto, Japan, stability of 100 ppm), which exhibits the maximum frequency drift around 125 °C. Pressure tests were then conducted on the sensor at 50 °C and 125 °C, with the test results shown in Figure 7f. For the scheme using direct frequency for calculation, its calculation error exhibits significant drift with temperature and has exceeded the original accuracy requirement. In contrast, the calculation error of the scheme using frequency-ratio for calculation is not affected by temperature variations and still meets the requirement of 0.02% FS. This scheme has a higher tolerance for the frequency stability of crystal oscillators and satisfies the demand for pressure measurement in high-temperature environments.

Due to fixture and circuit limitations, full-range calibration was not possible. Future work will include full-range validation after structural optimization. The sensitivity nonlinearity has been traced to fabrication issues; process improvements are expected to enhance linearity.

## 7. Conclusions

In this study, the V-shaped support beam and mass block structure successfully convert radial compressive stress into axial tensile stress, achieving a balance between pressure resistance and sensitivity. Meanwhile, this design also counteracts the frequency increase caused by the shorter beam length, reducing the requirements for the measurement circuit. Experimental results demonstrate that the sensor operates up to 175 MPa with sufficient sensitivity.

To address the conflict between high-temperature oscillators and measurement accuracy, a pressure-insensitive reference resonator was introduced, and a frequency-ratio computation scheme was established. This method eliminates the crystal oscillator frequency term, avoiding drift-induced errors. Experimental tests demonstrate that this pressure computation scheme is insensitive to the temperature drift of crystal oscillators, validating the feasibility of pressure computation under high-temperature conditions.

However, the three-resonator design poses challenges in chip miniaturization and fabrication yield. Although differential processing improves linearity, single-beam sensitivity still requires optimization. Future work will focus on structural miniaturization and process refinement to improve the linearity of sensitivity and enable broader application of the sensor.

## Figures and Tables

**Table 1:** Geometric parameters of sensor.

	Resonator I	Resonator II	Resonator III
main beam length	720 μm	720 μm	120 μm
main beam width	10 μm	10 μm	10 μm
support beam width	20 μm	20 μm	20 μm
support beam angle	56°	56°	/
support beam length	/	/	60 μm
mass beam length	400 μm	400 μm	515 μm
mass beam width	9 μm	10.5 μm	10 μm
width of the diaphragm structure	685 μm	484 μm	/

## Data Availability

The original contributions presented in the study are included in the article, further inquiries can be directed to the corresponding author.
